# The Associated Factors of Work Engagement, Work Overload, Work Satisfaction, and Emotional Exhaustion and Their Effect on Healthcare Workers: A Cross-Sectional Study

**DOI:** 10.3390/healthcare13020162

**Published:** 2025-01-16

**Authors:** Marina Moreno-Martínez, Iván Sánchez-Martínez

**Affiliations:** 1 Research and Innovation Unit, Primary and Community Care Management of Central Catalonia, 08242 Manresa, Spain; 2 Intelligence for Primary Care Research Group, Foundation University Institute for Research in Primary Care of Health Jordi Gol i Gurina, 08242 Manresa, Spain; 3 Faculty of Health Sciences, University of Manresa, 08242 Manresa, Spain; 4 Faculty of Health Sciences, International University of Valencia, 46002 Valencia, Spain; 5 Methodology, Methods, Models and Outcomes of Health and Social Sciences (M3O) Research Group, Faculty of Health Sciences and Welfare, Centre for Health and Social Care Research, University of Vic—Central University of Catalonia (UVIC-UCC), 08500 Vic, Spain; 6 Institute for Research and Innovation in Life Sciences and Health in Central Catalonia (IRIS-CC), 08500 Vic, Spain; 7 Navarcles Primary Care Center, Primary and Community Care Management of Central Catalonia, Catalan Institute of Health, 08270 Navarcles, Spain

**Keywords:** work engagement, work overload, work satisfaction, emotional exhaustion, healthcare workers

## Abstract

**Background:** In today’s fast-paced work environment, work engagement is crucial for both organizational success and individual well-being. **Objective:** Our aim is this study was to analyze the associated factors of work engagement, work overload, work satisfaction, and emotional exhaustion and describe their effect on nurses and physicians in the Central Catalonia Health Region during 2023. **Methods:** A multicenter cross-sectional study was conducted using an online questionnaire at the Territorial Management of Central Catalonia (Spain). The questionnaire was accessible from 28 November 2022 to 12 March 2023. The analysis was performed using the SPSS software. CHERRIES (Checklist for Reporting Results of Internet E-Surveys) guidelines were followed for communicating research results. **Results:** A total of 321 professionals answered the questionnaire, 60.7% of whom were nursing professionals and 39.3% of whom were medical professionals. Work overload, work satisfaction, and emotional exhaustion were associated with work engagement. Being a physician, permanent contracts, irregular work shifts, overtime, and salary were related to work overload and emotional exhaustion. Being a woman, salary, academic level, and irregular work shifts were related to work satisfaction. There was a gender inequality in work engagement among nursing professionals to the detriment of men. In terms of class inequality, there was a difference between occupational groups with respect to work overload and emotional exhaustion among women. **Conclusions:** Organizational practices need to be improved to promote greater engagement and work satisfaction, as well as to reduce emotional overload and exhaustion. This may include regulating unpaid overtime and promoting more stable working hours.

## 1. Introduction

In the current work environment, characterized by rapidly evolving professional demands and ever-changing organizational dynamics, work engagement has emerged as a fundamental pillar for organizational health and individual well-being. This is especially relevant for healthcare professionals, who play a crucial role in caring for the population. However, work engagement in the healthcare sector is often affected by work overload. High work demands, long hours, and constant pressure can lead professionals to feel overwhelmed, triggering mental and physical health problems. Likewise, healthcare professionals, due to the critical nature of their work, are exposed to significant work challenges that, sustained over time, can negatively influence their engagement [1,2].

Work engagement is considered a construct made up of vigor, dedication, and absorption. Vigor is characterized by enthusiasm and persistence when performing work tasks. Dedication refers to the involvement and sense of importance that the employee attributes to his or her job. Absorption corresponds to the degree to which a person immerses himself or herself in his or her work [3]. In this sense, work satisfaction is a crucial factor that can enhance or undermine work engagement. Healthcare professionals who enjoy a positive work environment tend to show higher levels of engagement. In this way, work satisfaction could act as a buffer against the negative effects of work overload, also keeping employees away from emotional exhaustion [4,5]. However, it should be noted that emotional exhaustion, or psychological burnout, is especially prevalent in the healthcare sector due to the demanding and often stressful nature of the work. When professionals experience emotional exhaustion, their ability to engage with their work decreases, which can lead to a lower quality of care [6,7]. In this regard, it is known that the reported data on burnout among healthcare workers are higher compared to the general population, with a difference of around 13%. Rates ranging from 21% to 67% more burnout are found, with the highest prevalence corresponding to that of community-based healthcare workers [7]. Therefore, it is important to emphasize that work engagement plays a significant protective role since it has been related to lower perceived stress at work and greater resilience and empathy [3].

Understanding how work engagement, work overload, work satisfaction, and emotional exhaustion are related can help in the development of strategies that promote work engagement and, therefore, improve the quality of patient care and the well-being of health professionals. For this reason, the hypotheses put forward in this study are that health professionals with high levels of job satisfaction and low levels of work overload and emotional exhaustion will have higher levels of job commitment, regardless of their sex or occupational class. In addition, job commitment, job satisfaction, work overload, and emotional exhaustion differ significantly between physicians and nurses, as well as between men and women, reflecting sex and occupational class differences in the health field. To answer this question, it was necessary to analyze the associated factors of work engagement, work overload, job satisfaction, and emotional exhaustion and to describe their effect on nurses and physicians in the Central Catalonia Health Region during 2023.

## 2. Materials and Methods

### 2.1. Design and Sample

A multicenter cross-sectional study was carried out through an online survey using the Microsoft Forms platform, a secure web application that meets international standards. The study was carried out through the Central Catalonia Territorial Management of the Catalan Institute of Health—a public company of Catalonia, Spain—which has 39 primary care centers and 872 structural positions, 438 of which are in medicine and 434 of which are in nursing [8]. A minimum necessary sample of 242 nursing and medicine professionals was calculated, with a confidence level of 95% and a precision of ±0.3 units, a population mean with values that are expected to have a standard deviation of around 2.5 units and an additional 20% of replacements. A convenience sample was carried out, sending a message with an invitation to participate in the study to the corporate email account. The message was sent through the people in charge of the mailing lists of the Communication Department of the Central Catalonia Territorial Management. The questionnaire was accessible from 28 November 2022 to 12 March 2023. CHERRIES (Checklist for Reporting Results of Internet E-Surveys) guidelines were followed for the communication of research results through online questionnaires [9].

### 2.2. Instruments and Variables

A self-administered questionnaire of 45 closed questions on a single page was used, divided into 5 parts: sociodemographic data (5 questions), occupational and work activity data (7 questions), work engagement (9 questions), work overload (5 questions), work satisfaction (10 questions), and emotional exhaustion (9 questions). The survey combined ad-hoc questions as well as others belonging to the following questionnaires.

Work Engagement: The Utrecht Work Engagement Scale (UWES-9) has 9 Likert-type response items. The score can range from 0 to 45 points, with 0 = never and 5 = always, with no cut-off points established, so the higher the score, the greater the commitment. The questionnaire was translated and validated into Spanish by Domínguez-Salas et al. 2022 with an α-Chronback of 0.93 for the entire scale [10].

Work Overload: Of the total number of questions in the Karasek work stress questionnaire, 5 were chosen, those related to the job demand category. The score range is between 5 and 20 points, corresponding to 1 = totally disagree and 4 = totally agree. The questionnaire was translated and validated into Spanish by Escribà-Agüir et al. 2001 with an α-Chronback of 0.74–0.88 [11].

Work Satisfaction: The Generic Job Satisfaction scale of MacIntyre and MacDonald is made up of 10 Likert-type questions (1 = never and 5 = always), with scores ranging between 10 and 50 points. The questionnaire was translated and validated into Spanish by Salessi et al. 2016 with an α-Chronback of 0.81 [12].

Emotional Exhaustion: Of the total number of questions in the Maslach Burnout Inventory (MBI) questionnaire, 9 were chosen, specifically those belonging to the emotional exhaustion category. The score range varies between 0 and 54 points, where 0 = never and 6 = every day. The cut-off points are set as follows: 0–18 = low, 19–26 = medium, and 27–54 = high. The questionnaire was translated and validated into Spanish by Forné et al. 2022 with an α-Chronback of 0.91 for the emotional exhaustion subscale [13].

### 2.3. Analysis

A bivariate descriptive analysis was performed, estimating the absolute and relative frequencies for qualitative variables and the mean and standard deviation for quantitative variables. The Kolmogorov–Smirnov test with Lilliefors correction was applied to all quantitative variables. Furthermore, the Student *t* test for independent samples was used for significance tests, together with the chi-square test, according to the nature of the variables. A multiple regression model was performed to determine the factors influencing work commitment, work overload, job satisfaction, and emotional exhaustion. The analysis was performed using SPSS version 26 for Windows (SPSS Inc., Chicago, IL, USA).

### 2.4. Ethical Considerations

The study was approved by the IDIAP Jordi Gol clinical research ethics committee with the registration code 22/205-P. All participants gave their consent to answer the questionnaire, where they were informed about the objective, the research team, and data management and storage, as well as the estimated response time. The data were anonymous and were recorded on the servers of the Territorial Management of Central Catalonia.

## 3. Results

A total of 321 nursing and medical professionals responded to the questionnaire, 60.7% of whom were nursing professionals and 39.3% of whom were medical professionals. Although the health sector is highly feminized, their distribution was asymmetrical, with female nurses accounting for 93.3% of the sample, while female physicians accounted for 76.2% (*p* = 0.000). Almost all physicians (94.5%) stated that their salary covered basic needs, while nurses did not reach three quarters (68.7%) (*p* = 0.000). In terms of contractual typology, 66.7% of physicians reported having a permanent contract, while the opposite is true for nurses, with 62.1% reporting having a temporary contract (*p* = 0.000). A total of 84.9% of physicians reported spending unpaid overtime at work, with an average of 5 h per week, and with this frequency being higher than that indicated by nurses (66.2% with an average of 3 h per week) (*p* = 0.000) (Table 1).

### 3.1. Factors Associated with Work Engagement

A multiple regression model was performed to predict the possible factors associated with work engagement. The model showed a good level of prediction (R = 0.738), with the independent variables explaining 54.4% of the variability of the work engagement variable, which was statistically significant with an F-test value of 0.000. The results showed a positive association between work overload and work engagement, with an increase of 0.501 units in work engagement for each one-unit increase in work overload (*p* < 0.001). This same relationship was observed with work satisfaction, where it was associated, on average, with an increase of 0.735 units in work engagement (*p* < 0.001). However, for the emotional exhaustion variable, a negative association was obtained, so that with a one-unit increase in emotional exhaustion, work engagement decreased by 0.246 units (*p* < 0.001). Regarding the sociodemographic variables, no statistically significant results were obtained (Table 2).

### 3.2. Factors Associated with Work Overload

The multiple regression analysis revealed a number of factors significantly associated with work overload. The model showed that medical professionals had more work overload than nursing professionals (B = 1.428, *p* < 0.001). Likewise, professionals with a permanent contract, rather than a temporary one, had 0.826 points more work overload (*p* < 0.05). However, those whose work shifts were irregular showed greater work overload, compared to workers with fixed work shifts (B = 0.554, *p* < 0.05). In addition, healthcare professionals who worked unpaid overtime had 1.467 points more work overload than their counterparts who did not (*p* < 0.001). On the other hand, those whose salaries covered their basic needs tended to experience less work overload (B = −0.342, *p* < 0.05). The model presented a prediction of level (R = 0.467, R2 = 0.218), with a statistically significant F-test = 0.000 (Table 2).

### 3.3. Factors Associated with Work Satisfaction

With regard to the variables predicting work satisfaction (R = 0.382, R2 = 0.146, F-test 0.000), the findings of the multiple regression analysis revealed gender as an influential factor. The results showed that women had more work satisfaction than men (B = 1.726, *p* < 0.05). With respect to academic level, professionals with a higher education level had less work satisfaction (B = −0.919, *p* < 0.05). Likewise, health workers whose work shifts were irregular showed 1835 points less work satisfaction compared to their colleagues with fixed work shifts (*p* < 0.01). On the other hand, those whose salaries covered their basic needs tended to experience 1541 points more work satisfaction (*p* < 0.001) (Table 2).

### 3.4. Factors Associated with Emotional Exhaustion

Regarding the factors influencing emotional exhaustion (R = 0.372, R2 = 0.139, F-test 0.000), it was observed that medical professionals had 3169 more points of emotional exhaustion than their nursing colleagues (*p* < 0.05). With respect to the type of contract, the findings showed that workers with a permanent contract reported greater emotional exhaustion, compared to those with a temporary employment contract (B = 4.783, *p* < 0.01). Likewise, healthcare workers with irregular work shifts reported 3374 more points of emotional exhaustion than those with fixed shifts (*p* < 0.01). In this sense, those who worked unpaid overtime had greater emotional exhaustion (B = 3.269, *p* < 0.05). On the other hand, health workers whose salaries covered their basic needs tended to experience 2666 fewer points of emotional exhaustion (*p* < 0.001) (Table 2).

### 3.5. Effect of Work Engagement, Work Overload, Work Satisfaction, and Emotional Exhaustion on Healthcare Professionals

In Table 3, firstly, healthcare professionals were stratified according to their occupational class, showing the results for the different variables based on sex, and thus eliminating the class effect. In this case, it was highlighted that work engagement was distributed in a statistically unequal manner. Gender inequality occurs among nursing professionals and to the detriment of men (*p* = 0.039). Thus, female nurses have more work engagement than their male counterparts. Secondly, healthcare professionals were also stratified according to sex, thus showing the results for the different variables based on occupational class. In this case, a pattern of class inequality was observed, with a difference between occupational groups in work overload and emotional exhaustion among women, but not among men (*p* = 0.000 and *p* = 0.006, respectively).

## 4. Discussion

The aim of this research was to analyze the associated factors of work engagement, work overload, work satisfaction, and emotional exhaustion and to describe their effect on nurses and doctors in the Central Catalonia Health Region during 2023. As to the composition of the sample, an asymmetric distribution was observed in terms of sex in the different occupational groups (*p* = 0.000). This fact coincides with other studies, where well-known vertical segmentation continues to be present in the health sector, despite it being a highly feminized sector [14,15,16].

With regard to the factors associated with work engagement, the findings indicated that health workers who have more work overload are also those who are more committed to their work. Work satisfaction also had a positive relationship with work engagement, while the association with emotional exhaustion was negative. This relationship has also been observed in studies by other authors, where work overload, work satisfaction, and emotional exhaustion were significantly related to work engagement [1,2]. This association could be explained by the job demands–resources (JD-R) theory of Arnold Bakker et al. According to this model, job demands can have a positive effect on work engagement, as workers can feel motivated and stimulated by these demands, leading to greater involvement and dedication in their work. On the other hand, demands can also lead to burnout, which in turn is negatively related to work engagement. In this way, burnout decreases work engagement because exhausted employees lack the energy needed to engage in their tasks. Workers with high levels of burnout may feel that they do not have the emotional resources to fully dedicate themselves to their work, displaying, for example, decreased dedication, a negative attitude towards tasks, and emotional disengagement from work. In this same line of thought, job satisfaction is positively associated with work engagement, because when employees are satisfied with their work, they are more likely to feel motivated. For this reason, satisfaction provides a positive environment that fosters vigor, dedication, and absorption, helping workers to get involved with the organization’s goals [17].

As for the factors associated with work overload and emotional exhaustion, the same statistically significant variables were obtained, so the point is discussed together. The results showed that doctors were the ones who had the greatest overload and exhaustion. This coincides with the findings of other studies, where medical professionals are the ones who have the most unfavorable exposures to high quantitative demands, which can lead to greater exhaustion [14,15]. A relationship was also observed with the type of contract, work shifts, and overtime. The latest European survey on working conditions showed that more than half of employees with permanent contracts worked at high speed (51.8%). In addition, they had to work under very tight deadlines, a difference of around 9% compared to workers with temporary contracts [18]. This could be because permanent contracts, while providing stability and job security, also entail higher expectations in terms of productivity and commitment, and workers may feel pressured to take on more tasks and responsibilities. This can lead to a backlog of work and greater burnout, as these types of employees have more defined long-term roles. In this sense, irregular shifts make it difficult to plan personal and family time, which can increase stress and the feeling of being emotionally overloaded and exhausted. In addition, irregular shifts lead to an imbalance between work and personal life, making workers feel that they must always be available for work [19]. Likewise, several authors have linked rotating shifts with presenteeism and exhaustion, as well as long working hours (overtime) [20,21]. On the other hand, salary was negatively associated with work overload and emotional exhaustion, such that those whose salary was insufficient to cover their needs tended to have greater work overload and exhaustion. This scenario was also corroborated by the study by Salas-Nicas et al. where workers with lower salaries are more exposed to high strain (high demands and low control to cope with them) with a difference of around 14%, compared to those with a salary sufficient to cover their needs [22].

In relation to the factors associated with job satisfaction, the results indicated that being a person whose salary allowed them to cover their needs and being a woman were positively related to satisfaction. However, a high level of education and irregular shifts were negatively associated with satisfaction. These results coincided with those published by Elsahoryi et al. where workers with higher monthly salaries were 1.53 times more likely to have higher job satisfaction compared to those with lower monthly salaries [23]. This was also confirmed by Singh et al., where job satisfaction was higher for those who received higher amounts of remuneration, although a wage gap was found to the detriment of women (*p* < 0.001) [24]. Despite the obvious wage gap, women are more likely than men to respond positively in terms of job satisfaction [23,25,26]. This could be due to different expectations between men and women due to cultural and social factors. While one of the most important factors for men when considering a new job is monetary compensation, women tend to value work–life balance or flexibility, as well as a good work environment among other aspects. In addition, if women’s initial expectations about their remuneration are lower, they may feel greater satisfaction when these expectations are met or exceeded, even though they objectively earn less [26]. In relation to educational level, the research by Li et al. showed that professionals with a higher educational level had less job satisfaction. A possible explanation would be that people with a higher educational level tend to have higher expectations regarding their jobs, both in terms of salary, development opportunities, and personal satisfaction. In addition, another reason could be due to the phenomenon known as “underemployment”. This situation occurs when the employee is overqualified for the job he or she performs, which generates a mismatch of skills that creates dissatisfaction due to the feeling of not fully utilizing his or her skills and knowledge [4]. However, it must be taken into account that work satisfaction is a multidimensional construct that can be influenced by several factors. Ran et al. did not find statistical significance between academic level and job satisfaction, but did relate it to the intention to leave the job [27]. Along the same lines, with respect to work shifts, evidence points to irregular shifts as predictors of greater job dissatisfaction, compared to regular shifts [28], as well as the duration of the shift, where longer shifts lead to lower job satisfaction [29]. Irregular schedules can disrupt sleep and general health [30,31], and also can lead to physical and mental health problems such as fatigue and stress [28].

The axes of segregation in the labor market did not go unnoticed in this study. On the one hand, a pattern of inequality in occupational class was obtained, finding class differences only in women for work overload and emotional exhaustion, with these results being more unfavorable among medical professionals than among nursing professionals. On the other hand, the effect of work engagement on sex—eliminating the effect of occupational class—highlighted a statistically significant unequal distribution to the detriment of male nurses, with female nurses being more committed to their work. These results are inconsistent with those found in the literature. On the one hand, Rivera et al. did not find statistically significant differences between sex and work engagement [32]. However, Wang et al. showed that work engagement was higher among men compared to women (*p* < 0.001) [33]. The relationship between work engagement and gender is complex, as it depends on the cultural, social, and economic context. For this reason, it cannot be conclusively stated that one gender has less work engagement than another. However, it should be noted that there are some factors that influence how work engagement is perceived according to gender. For example, women have traditionally taken on a greater share of the responsibilities for caring for the home and children. This may lead some women to have to combine these responsibilities with their work, which is sometimes wrongly interpreted as a lack of work engagement. However, many women work as hard as their male counterparts but have to divide their time between work and home. This conflict often results in the so-called glass ceiling, where women may face barriers that affect their professional development and incorrectly interpret this as a lack of commitment when, in reality, it is a reaction to a work environment that does not support their growth. The perceived differences are often the result of external and systemic factors rather than an intrinsic lack of commitment. For this reason, it is important to consider the various moderating factors that may be influential when analyzing work engagement from a gender perspective [34].

We believe that the results of our research make a significant contribution to the literature. However, there are several limitations in the study that could affect the robustness of the results, so we describe them in the following section.

### Limitations

It should be noted that this study has some limitations. Given the geographical extent of the study, an online questionnaire was chosen, which was the best option available for this case and is also used by international organizations (Eurofound). As it was an anonymous questionnaire, it was not possible to ensure that each person responded only once. Furthermore, the response rate could not be estimated, since the number of workers who opened the email inviting them to participate and the number of professionals who received the link to the snowball questionnaire are unknown. Even so, the number of participants exceeded the sample size initially calculated. A reminder message inviting them to participate was also sent as a method of increasing the response rate [35]. Likewise, to improve the representativeness of the sample, all doctors and nurses with any type of employment contract were included, although it is possible that not all professionals with temporary contracts frequently checked their institutional email.

## 5. Conclusions

The results of this study point to several factors that need to be addressed. Organizational practices need to be improved to promote greater commitment and work satisfaction, as well as to reduce work overload and emotional exhaustion. Therefore, the implementation of policies that manage the balance between workload and available resources should be considered. This may include regulating unpaid overtime and promoting more stable work schedules. In addition, it is essential to foster a work environment that increases job satisfaction, which can increase work engagement. These strategies would not only improve the well-being of health professionals but could also translate into better patient care by promoting more committed and less emotionally exhausted staff. For future research, qualitative studies would be necessary to explain the quantitative results and answer the origin of work overload and emotional exhaustion. On the other hand, intervention studies would be needed to increase job satisfaction, as well as commitment to the organization.

## Figures and Tables

**Table 1:** Percentages of occupations in relation to socio-demographic and occupational variables.

	Medical Professionals (n = 126)	Nursing Professionals (n = 195)	*p*-Value
Sex (N(%))			
Male	30 (23.8)	13 (6.7)	0.000
Female	96 (76.2)	182 (93.3)	
$\mathrm{Age}\;(\bar{x}$ ± SD)	46 ± 11	45 ± 10	0.294
Dependents in their charge (N(%))	57 (45.2)	105 (53.8)	0.132
Academic level (N(%))			
Degree	35 (27.8)	72 (36.9)	0.000
University Master’s	24 (19)	114 (58.5)	
Specialist	66 (52.4)	8 (4.1)	
Doctorate	1 (0.8)	1 (0.5)	
Salary covering basic needs (N(%))	119 (94.5)	134 (68.7)	0.000
Type of contract (N(%))			
Permanent	84 (66.7)	74 (37.9)	0.000
Temporary	42 (33.3)	121 (62.1)	
Rotating or irregular work shift (N(%))	55 (43.7)	89 (45.6)	0.726
Position held in the company (N(%))			
Assistance	113 (89.7)	174 (89.2)	0.898
Direction, management	13 (10.3)	21 (10.8)	
Unpaid overtime work	107 (84.9)	129 (66.2)	0.000
$\mathrm{Average}\;\mathrm{weekly}\;\mathrm{hours}\;\mathrm{of}\;\mathrm{unpaid}\;\mathrm{overtime}\;\mathrm{work}\;(\bar{x}$ ± SD)	5 ± 6	3 ± 5	0.000
$\mathrm{Years}\;\mathrm{of}\;\mathrm{service}\;\mathrm{in}\;\mathrm{the}\;\mathrm{company}\;(\bar{x}$ ± SD)	16 ± 11	13 ± 10	0.072

**Table 2:** Factors associated with work engagement, work overload, work satisfaction, and emotional exhaustion.

	Unstandardized Coefficient β (95% CI)			
	Work Engagement	Work Overload	Work Satisfaction	Emotional Exhaustion
Work overload	0.501 (0.232, 0.770) ***	x	x	x
Work satisfaction	0.735 (0.600, 0.869) ***	x	x	x
Emotional exhaustion	−0.246 (−0.310, −0.183) ***	x	x	x
Sex	−0.367 (−2.140, 1.406)	0.169 (−0.656, 0.994)	1.726 (0.002, 3.450) *	−1.113 (−5.029, 2.803)
Age	0.028 (−0.063, 0.119)	−0.020 (−0.062, 0.023)	0.035 (−0.053, 0.124)	−0.149 (−0.351, 0.053)
Dependents in their charge	0.319 (−0.862, 1.499)	0.211 (−0.340, 0.761)	−0.372 (−1.522, 0.778,)	2.528 (−0.085, 5.140)
Academic level	0.546 (−0.304, 1.397)	0.159 (−0.237, 0.554)	−0.919 (−1.746, −0.092) *	0.616 (−1.262, 2.494)
Salary covering basic needs	−0.576 (−1.246, 0.095)	−0.342 (−0.645, −0.040) *	1.541 (0.909, 2.173) ***	−2.666 (−4.103, −1.230) ***
Occupational group	−0.969 (−2.437, 0.498)	1.428 (0.765, 2.091) ***	0.573 (−0.813, 1.959)	3.169 (0.021, 6.318) *
Type of contract	−0.583 (−2.194, 1.027)	0.826 (0.082, 1.570) *	−0.500 (−2.055, 1.055)	4.783 (1.251, 8.316) **
Rotating or irregular work shift	0.698 (−0.478, 1.873)	0.554 (0.013, 1.095) *	−1.835 (−2.965, −0.705) **	3.374 (0.806, 5.941) **
Position held in the company	0.485 (−1.650, 2.621)	0.630 (−0.364, 1.625)	1.158 (−0.920, 3.237)	−1.270 (−5.992, 3.452)
Unpaid overtime work	0.843 (−0.656, 2.342)	1.467 (0.783, 2.150) ***	−0.936 (−2.364, 0.493)	3.269 (0.24, 6.514) *
Average weekly hours of unpaid overtime work	0.061 (−0.074, 0.196)	0.007 (−0.056, 0.071)	0.015 (−0.117, 0.147)	0.078 (−0.222, 0.379)
Years of service in the company	−0.021 (−0.118, 0.077)	−0.036 (−0.081, 0.010)	−0.023 (−0.118, 0.072)	0.000 (−0.216, 0.215)

* *p* < 0.05; ** *p* < 0.01; *** *p* < 0.001.

**Table 3:** $\bar{X}$ ± SD of work engagement, work overload, work satisfaction, and emotional exhaustion by sex for each occupational group, and by the occupational group for each sex.

	Medical Professionals		*p*-Value	Nursing Professionals		*p*-Value
	Male	Female		Male	Female	
Work engagement	26.57 ± 9.35	26.16 ± 6.60	0.586	22.23 ± 9.58	27.32 ± 7.40	0.039
Work overload	17.50 ± 2.84	18.20 ± 2.25	0.317	16.85 ± 1.57	16.31 ± 2.74	0.566
Work satisfaction	32.83 ± 6.72	34.23 ± 5.02	0.420	30.77 ± 7.00	33.75 ± 5.19	0.131
Emotional exhaustion	35.90 ± 14.50	36.53 ± 11.37	0.911	37.15 ± 10.89	32.12 ± 12.09	0.111
	**Male**		***p*-value**	**Female**		***p*-value**
	**Medical professionals**	**Nursing professionals**		**Medical professionals**	**Nursing professionals**	
Work engagement	26.57 ± 9.35	22.23 ± 9.58	0.159	26.16 ± 6.60	27.32 ±7.40	0.166
Work overload	17.50 ± 2.84	16.85 ± 1.57	0.222	18.20 ± 2.25	16.31 ±2.74	0.000
Work satisfaction	32.83 ± 6.72	30.77 ± 7.00	0.440	34.23 ± 5.02	33.75 ± 5.19	0.725
Emotional exhaustion	35.90 ± 14.50	37.15 ± 10.89	0.969	36.53 ± 11.37	32.12 ± 12.09	0.006

## Data Availability

The data are stored on the servers of the Territorial Management of Central Catalonia and are not publicly available for ethical reasons. The informed consent did not include the transfer of data to third parties or international data transfer.

## Abbreviations

The following abbreviations are used in this manuscript:

UWES-9	The Utrecht Work Engagement Scale
MBI	Maslach Burnout Inventory
